# Multimodal chemoradiotherapy including interstitial brachytherapy enhances outcomes in FIGO stage IVA cervical cancer: a focus on tumor control and quality of life

**DOI:** 10.1007/s00066-025-02407-x

**Published:** 2025-05-21

**Authors:** Maria Neu, Carolin Michaela Wöhrl, Renate Walter, Nikolaos Balagiannis, Christoph Poettgen, Lukas Käsmann, Martin Stuschke, Christian Dannecker, Georg Stüben, Klaus-Henning Kahl

**Affiliations:** 1 Department of Radiotherapy and Radiation Oncology, Klinik für Strahlentherapie und Radioonkologie, Faculty of Medicine, University of Augsburg, Stenglinstr. 2, 86156 Augsburg, Germany; 2 Comprehensive Cancer Center Augsburg (CCCA), Faculty of Medicine, University of Augsburg, 86156 Augsburg, Germany; 3 Comprehensive Cancer Center Alliance WERA (CCC WERA), 86156 Augsburg, Germany; 4 Bavarian Cancer Research Center (BZKF), 86156 Augsburg, Germany; 5 Department of Radiation Protection and Medical Physics, Faculty of Medicine, University of Augsburg, 86156 Augsburg, Germany; 6 Department of Radiation Therapy, West German Cancer Center (WTZ), University Hospital Essen, Essen, Germany; 7 Department of Radiation Oncology, University Hospital, LMU Munich, Munich, Germany; 8 German Cancer Consortium (DKTK), Partner Site Munich, 80336 Munich, Germany; 9 Bavarian Cancer Research Center (BZKF), Munich, Germany; 10 Department of Gynecology and Obstetrics, Faculty of Medicine, University of Augsburg, 86156 Augsburg, Germany

**Keywords:** Locally advanced cervical cancer, High-dose-rate brachytherapy, Image-guided radiotherapy, Bladder or rectal infiltration, Patient-reported outcome measures

## Abstract

**Purpose:**

This study was performed to evaluate the outcomes of advanced radiotherapy techniques, including image-guided adaptive brachytherapy (IGABT), in International Federation of Gynecology and Obstetrics (FIGO) stage IVA cervical cancer patients with adjacent organ infiltration. A further aim was to identify prognostic factors influencing overall survival (OS) and local control (LC) in these patients, with a particular focus on toxicity and patient-reported outcomes (PROs).

**Methods:**

This retrospective, single-center study included 31 patients with FIGO stage IVA cervical cancer treated with definitive chemoradiotherapy between 2010 and 2020. All 31 patients underwent external-beam radiotherapy (EBRT), with concurrent cisplatin-based chemotherapy (CTX) administered in 25 cases and additional high-dose-rate brachytherapy (BT) performed in 24 cases. Treatment-related adverse events were categorized in accordance with the Common Terminology Criteria for Adverse Events (CTCAE; version 5.0) [1]. PROs were evaluated using the European Organization for Research and Treatment of Cancer Quality of Life Questionnaire version 3.0 (EORTC QLQ-C30), while sexual function was assessed through three specific questions adapted from the EORTC QLQ-BR23 module.

**Results:**

Median OS was estimated at 51.7 months, with 2‑ and 5‑year OS rates of 58.1 and 46.2%, respectively. Median progression-free survival (PFS) was 48.1 months (95% CI: 0–96.2 months), with 2‑ and 5‑year PFS rates of 52 and 37%. The 10-year LC probability was 70.4%, showing a significant association with improved OS (*p* = 0.0039). Eastern Cooperative Oncology Group (ECOG) performance status (*p* = 0.014) and nodal involvement were identified as prognostic factors. The estimated median OS was 108 months for patients treated with BT and 51.7 months for those without. Patients receiving six fractions or a cumulative BT dose of ≥ 24 Gy demonstrated improved 5‑year OS rates of 62.3%, although the difference was not statistically significant. Acute toxicities were reported in 83.9% of patients, primarily grades 1–2, with severe complications such as fistula formation occurring in 16.1%. Late toxicities, predominantly affecting the gastrointestinal and urogenital systems, were observed in 45.2% of patients. Patient-reported outcomes indicated mild to moderate impairments of quality of life, with fatigue and gastrointestinal symptoms being the most frequently reported issues.

**Conclusion:**

Advanced radiotherapy, particularly IGABT, achieves durable LC in patients with FIGO stage IVA cervical cancer, supporting its use as a cornerstone of curative-intent treatment. However, systemic progression remains a major challenge, highlighting the need for novel therapeutic strategies, including immunotherapy and liquid biopsy for treatment monitoring. Future prospective trials are essential to validate these findings and refine therapeutic protocols, particularly for high-risk subgroups. Ensuring equitable access to these advanced treatments is critical for improving global outcomes in cervical cancer care.

## Introduction

Cervical cancer is one of the most commonly diagnosed cancers in women on a global scale, ranking fourth in terms of both prevalence and mortality [2]. In the context of early-stage cervical cancer, radical surgery continues to represent an efficacious therapeutic approach. In contrast, for patients with unresectable locally advanced cervical cancer (LACC), platinum-based concurrent chemoradiotherapy (CCRT) represents the standard treatment option. Nevertheless, in cases of locally advanced disease, particularly International Federation of Gynecology and Obstetrics (FIGO) stage IVA, which accounts for approximately 3.1% of cases, there remains a significant therapeutic challenge due to the limited prognosis and survival rates.

Historically, the presence of bladder or rectal involvement in LACC has often been associated with predominantly palliative treatment approaches. However, advancements in radiation oncology have shifted this perspective, enabling the pursuit of curative strategies in selected cases. The current standard of care for such complex scenarios combines intensity-modulated external-beam radiotherapy (EBRT), concurrent platinum-based CT, and image-guided adaptive brachytherapy (IGABT). This multimodal approach facilitates precise dose escalation to the tumor while minimizing radiation exposure to surrounding healthy tissues, thereby improving both therapeutic outcomes and patient quality of life [3, 4].

The objective is to evaluate the efficacy of an intensified multimodal treatment concept, with a particular focus on local control and long-term survival in this specific patient group. By means of a detailed statistical analysis, we seek to identify factors that predict treatment success and provide guidance for future therapeutic decisions in cases involving bladder or rectal infiltration.

Modern magnetic resonance imaging (MRI)-based treatment planning allows precise target definition and adaptation to tumor changes during therapy. These technical improvements provide treatment options for patients who previously had limited therapeutic choices and may evade deterioration in quality of life (QoL). Despite these advances, there is still a lack of standardized treatment protocols for patients with organ infiltration, and recommendations of evidence-based guidelines remain limited.

## Materials and methods

### Study design and participants

This study was approved by the Ethics Committee of the Ludwig Maximilian University Munich (approval number: 21-0603), and all participants provided written consent for the use of their clinical data in this research.

In this monocentric retrospective study, we evaluated all patients with FIGO stage IVA cervical cancer consecutively treated with chemoradiotherapy at our institution between January 1, 2010, and December 31, 2020. The follow-up period extended to December 31, 2023. All patients had biopsy-proven cervical cancer and evidence of bladder or rectal involvement. They were treated with a three-pronged approach comprising EBRT, concurrent cisplatin-based chemotherapy, and high-dose rate brachytherapy (BT). The evaluation of patient-reported outcomes (PROs) assessing quality of life was conducted using the European Organization for Research and Treatment of Cancer Quality of Life of Cancer Patients Questionnaire version 3.0 ([5], EORTC-QLQ-C30). Treatment-related adverse events were graded in accordance with the Common Terminology Criteria for Adverse Events (CTCAE), version 5.0 [1]. The assessment of sexual function was conducted via three questions derived from the EORTC QLQ-BR23 module [6].

### Statistical analysis

Descriptive statistics were employed to summarize patient characteristics and treatment parameters, including measures of central tendency (mean, median) and variability (range). Frequency distributions were expressed as absolute numbers (*n*) and percentages (%). Outcomes were estimated using Kaplan–Meier methods, with comparisons between subgroups performed using log-rank tests. The significance level was set to 0.05. For our analyses, a “month” was standardized to 30.42 days, with the index date defined as the last day of radiotherapy, either EBRT or BT. The survival analyses were performed using the R programming language (version 3.5.7; R Foundation for Statistical Computing, Vienna, Austria). The analyses employed the survival (version 3.5.7) and survminer (version 0.5.0) packages, which are specifically designed for survival data analysis and visualization. Patients with incomplete mortality data were censored in survival analyses.

## Results

### Baseline characteristics

A total of 31 patients with FIGO stage IVA cervical cancer, all presenting with adjacent organ infiltration, were included in the analysis and treated with curative-intent chemoradiotherapy. Among them, 25 patients (80.6%) had bladder infiltration, including 11 patients (35.5%) with isolated bladder involvement. Rectal infiltration was observed in 20 patients (64.5%), of whom 6 patients (19.4%) had isolated rectal involvement. Notably, 14 patients (45.2%) exhibited infiltration of both bladder and rectum. The median age of the cohort was 52 years (range 20–79 years). A total of 93.5% of patients had squamous cell carcinoma, with two patients exhibiting adenocarcinoma. Regarding regional nodal status, 10 patients (32.3%) had no evidence of nodal involvement, whereas 21 patients (67.7%) were found to have tumor-positive lymph nodes. For further details, please refer to Table 1, which provides a comprehensive overview of patient characteristics.

**Table 1:** Patient characteristics

	*n*	%
*Age at diagnosis*		
< 50 years	14	45.2
≥ 50 years	17	54.8
*Tumor histology*		
Squamous cell carcinoma	29	93.5
Adenocarcinoma	2	6.5
*Tumor size (bulky disease)*		
≤ 4 cm	–	–
> 4 cm	31	100
*Nodal involvement*		
None	10	32.3
Pelvic	12	38.7
Pelvic + paraaortic	9	29.0
*Infiltration*		
Bladder	11	35.5
Rectal	6	19.4
Bladder and rectal	14	45.2
Other structures (e.g., pelvic wall, ureter, urethra)	7	22.6
*ECOG*		
0	20	64.5
1	11	35.5

*ECOG* Eastern Cooperative Oncology Group performance status

### Treatment protocols

All patients underwent pelvic EBRT at a median dose of 50.4 Gy, delivered using either three-dimensional conformal radiotherapy (3D-CRT) or intensity-modulated radiotherapy (IMRT). The clinical target volume (CTV) was delineated in accordance with established guidelines using magnetic resonance imaging (MRI)-based treatment planning [7]. Brachytherapy commenced at approximately the fifth week of EBRT, which had delivered a dose of approximately 15–30 Gy. The treatment was delivered in 3–6 fractions of 3D IGABT, once a week, with a prescribed dose of 4–5 Gy. Chemotherapy was administered concurrently with EBRT, comprising intravenous medication once weekly for a period of 5–6 weeks.

### Percutaneous radiotherapy

External-beam radiotherapy was administered to all 31 patients, with 23 (74.2%) receiving a total dose of 50.4 Gy (range 45–70.4 Gy). A boost dose was administered to tumor-involved pelvic lymph nodes in 13 patients (41.9%), with a median boost of 59.4 Gy (range 59.4–70 Gy). The majority of patients (83.9%) received a single dose of 1.8 Gy, while 5 patients (16.1%) received a reduced single dose of 1.6 Gy in order to mitigate the risks associated with advanced infiltration and fistula complications. Extended-field radiotherapy to the paraaortic lymph nodes was performed in 9 patients (29%) with paraaortic involvement. The median duration of EBRT was 6.1 weeks (range 5.1–10.3 weeks), with longer periods observed in cases where the dose was escalated to 70.4 Gy.

### Interstitial brachytherapy

Of the 31 patients, 24 (77.4%) received interstitial BT in addition to EBRT. Brachytherapy was performed under general anesthesia with prophylactic antibiotics, in accordance with established techniques [8], using CT (Siemens Somatom Sensation, Siemens AG, Medical Solutions, 91301 Forchheim, Germany) image guidance for interstitial high-dose-rate (HDR) BT and delivered a median total dose of 20 Gy (range 12–24 Gy) in 4 fractions (range 3–6). Target volume definition and reporting were according to gynecological (GYN) GEC-ESTRO recommendations [9]. The median dose coverage (V100) of the planning target volume was 98.1%, with a median tumor volume of 149 ccm (range 52–260 ccm). One patient did not undergo the planned BT procedure due to a rapid tumor response during EBRT, leading to a fistula increase.

### Concurrent chemotherapy

Concurrent chemotherapy (CTX) was administered to 25 patients (80.6%), with cisplatin as the standard regimen in 18 cases (58.1%), typically at 40 mg/m^2^ per week. Mitomycin C was employed in 2 patients (6.5%), while 5 patients (16.1%) required modifications to their regimen due to toxicity or other factors. A total of 6 patients (19.4%) did not receive CTX for medical or personal reasons. A summary of details pertaining to EBRT, BT, and CTX can be found in Table 2.

**Table 2:** Treatment details

Modality	*n*	%	Gy	Weeks	Range
*EBRT n* *=* *31*					
Median total dose	31	100	50.4	–	45.0–70.4
Median duration of EBRT	–	–	–	6.1	5.1–10.3
Median single dose	26	83.9	1.8	–	1.6–1.8
Pelvic boost	13	41.9	59.4	–	59.4–70
Median CTV volume pelvic (ccm)	773	–	–	–	300–1611
Extended-field paraaortic	9	29.0	50.4	–	–
Median CTV volume paraaortic (ccm)	272	–	–	–	122–390
*BT n* *=* *24*					
Median total dose	–	–	20	–	15–30
Median single dose	–	–	4	–	4–5
Median fractionation	4	–	–	–	3–6
Median dose coverage %	–	98.1	–	–	85.1–99.9
Median CTV volume (ccm)	149	–	–	–	52–260
*CTX n* *=* *25*					
Cisplatin	18	72	–	5	5–6
Mitomycin C	2	8	–	–	–

*EBRT* external beam radiotherapy, *CTV* clinical target volume, *BT* brachytherapy, *CTX* chemotherapy, *ccm* cubic centimeter, *Gy* Gray

### Diagnostic and therapeutic interventions

Of the total patient collective, 17 (54.8%) underwent diagnostic or therapeutic interventions prior to commencing chemoradiotherapy. The primary objectives of these procedures were disease staging and treatment preparation. Of these procedures, the most prevalent was pelvic or paraaortic lymphadenectomy (LNE), which was performed in 15 patients for the purpose of staging. In the remaining cases, nodal status was assessed using imaging modalities such as positron-emission tomography–computed tomography (PET-CT) or MRI. Adnexectomy was performed in 12 patients, while 5 patients underwent uterine curettage. One patient underwent ovarian transposition to safeguard ovarian function from radiation effects, while cervical conization was also included in one diagnostic workup. Finally, the remaining 14 patients (45.2%) did not undergo any invasive procedures prior to treatment.

### Treatment outcomes

#### Overall survival (OS)

**Fig. 1:**
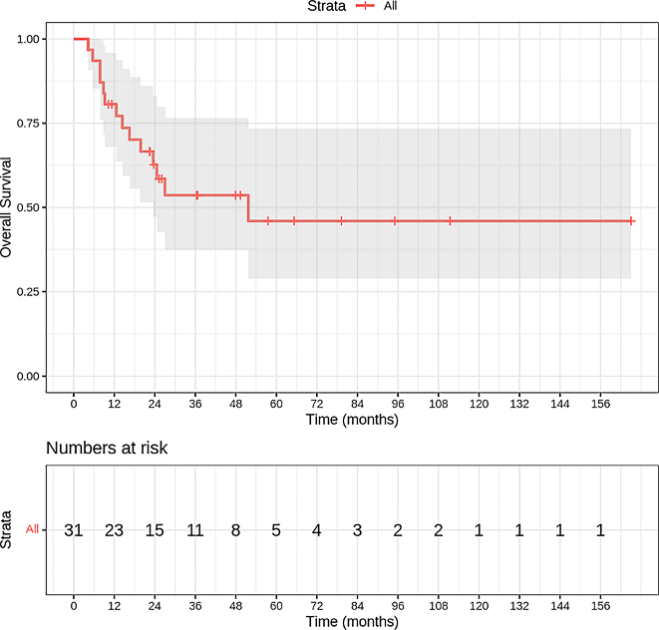
Kaplan–Meier curve showing overall survival of the entire cohort

Survival analysis of the 31-patient cohort revealed a temporal pattern of declining survival probabilities, from 94.0% at 4.3 months to 46.2% at 5 years after treatment. The cohort reached median survival at 51.7 months, with survival rates of 62.2 and 53.3% at 2 and 3 years, respectively. Tumor-related mortality occurred in 14 patients (45.2%), exclusively within the first 51.7 months of follow-up, while 17 patients (54.8%) remained alive or were lost to follow-up. The substantial proportion of censored cases necessitates careful interpretation of the median survival estimate. The temporal evolution of overall survival is illustrated in Fig. 1.

#### Progression-free survival (PFS)

**Fig. 2:**
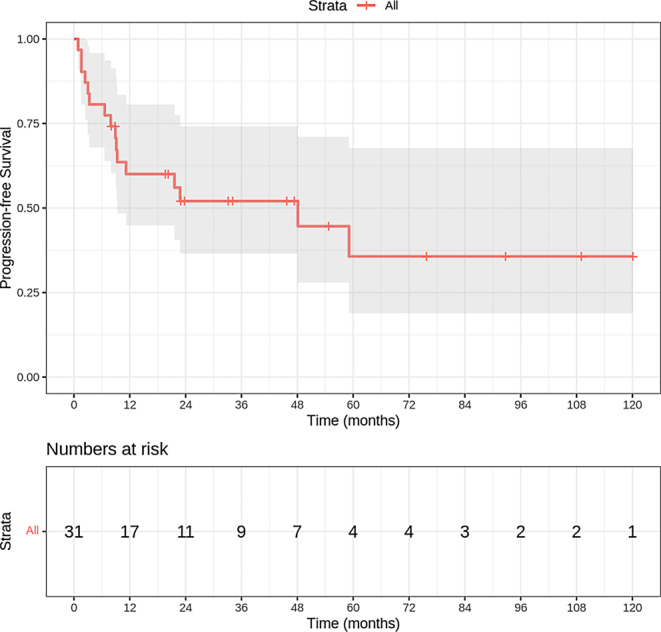
Progression-free survival rate

The median PFS was 48.1 months (95% confidence interval [CI]: 0–96.2 months). The 2‑ and 5‑year PFS probabilities were 52 and 37%, respectively. By the end of the observation period, 16 events (progression or death) had occurred, while 15 patients (48.4%) were censored (Fig. 2).

#### Local control and patterns of disease recurrence

**Fig. 3:**
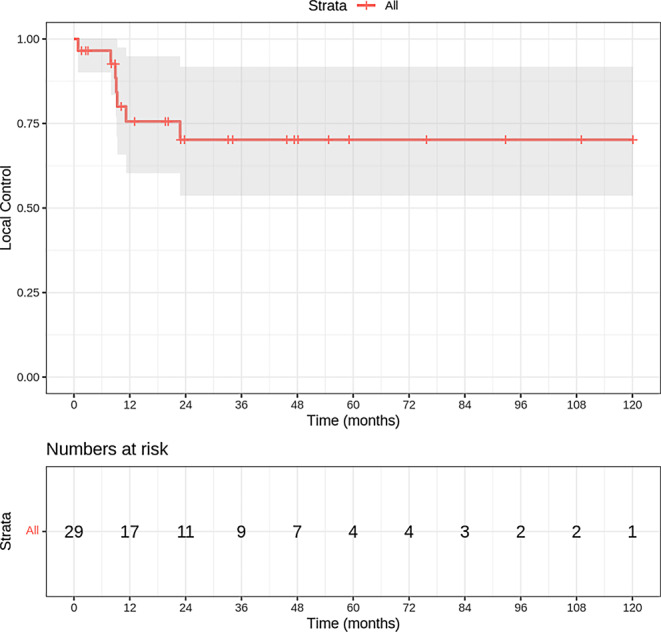
Local control rate

In our cohort, 22 out of 29 evaluable patients (75.9%) achieved local tumor control following chemoradiotherapy, with 14 patients (63.6%) achieving complete remission and 4 patients (18.2%) maintaining stable pelvic disease. Kaplan–Meier analysis yielded local control probabilities at 2, 5, and 10 years of 70.4% (Fig. 3). Among the 7 patients (24.1%) who experienced local recurrence, 2 (6.9%) had recurrence at the cervix, while 4 (13.8%) presented with pelvic lymph node metastases, including 2 cases involving the paraaortic lymph nodes. Furthermore, residual pelvic disease was observed to have progressed in 3 patients (10.3%), all of whom exhibited tumor extension into adjacent organs. Distant metastases were observed in 5 patients (22.7%), with the lungs or pleura being the primary site, followed by the liver, peritoneum, and bones. Two patients were excluded from the analysis due to the inability to ascertain their status at the follow-up timepoint.

### Prognostic factors

**Fig. 4:**
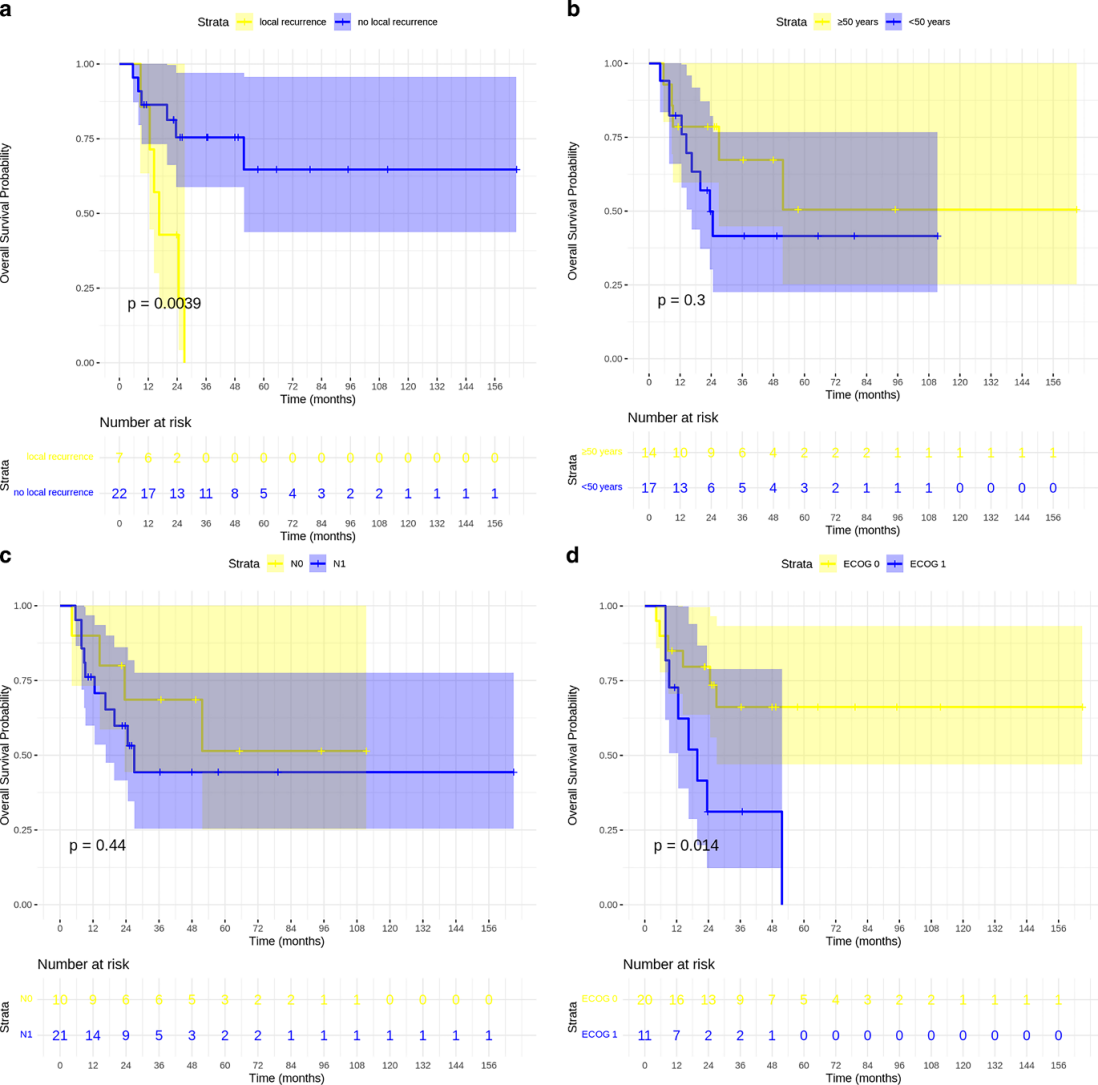
Overall survival (OS) based on key prognostic factors is illustrated in a–d: OS by local control (**a**), OS by patient age (**b**), OS by lymph node status (**c**), OS by ECOG performance status (**d**)

Achievement of local control emerged as a significant predictor of overall survival (*p* = 0.0039). Patients maintaining local control (*n* = 22) demonstrated superior outcomes, with mean overall survival of 115.7 months (95% CI: 81.99–149.45) and 2‑ and 5‑year survival probabilities of 75.2 and 65.1%, respectively. Notably, 81.8% (18/22) of these patients survived beyond 5 years. Conversely, patients experiencing local failure (*n* = 7) exhibited substantially diminished survival, with a median OS of 16.5 months (95% CI: 11.1–21.9), with only 1 patient surviving beyond 5 years (Fig. 4a).

Stratification of survival outcomes by age (< 50 versus ≥ 50 years) revealed distinct survival patterns, albeit not reaching statistical significance (*p* = 0.3). The younger cohort (*n* = 14) demonstrated more favorable outcomes, with a mean survival of 96.8 months (95% CI: 50.5–143.0) and 2‑ and 5‑year survival probabilities of 78.0 and 51.2%, respectively. The mortality rate in this group was 35.7% (5/14). In contrast, patients ≥ 50 years (*n* = 17) exhibited a shorter median survival of 23.6 months (95% CI: 15.8–31.2) and a mean survival of 55.2 months (95% CI: 30.8–79.6), with 2‑ and 5‑year survival rates of 49.0 and 40.9%, respectively. The mortality rate in the older cohort reached 52.9% (9/17) (Fig. 4b).

Initial lymph node status was evaluated as a prognostic factor. Among patients with N0 status (*n* = 10), 40% died during the observation period, whereas in the N1 group (*n* = 21), 47.6% succumbed to the disease. The 2‑ and 5‑year OS probabilities were 69.3 and 53.9% for N0 patients, compared to 58.6 and 44.3% for N1 patients. Kaplan–Meier analysis revealed a trend toward better survival in patients without lymph node involvement; however, the difference in survival distributions between the groups was not statistically significant (*p* = 0.44; Fig. 4c).

Analysis of survival outcomes stratified by Eastern Cooperative Oncology Group (ECOG) performance status demonstrated significant prognostic implications (*p* = 0.014). Patients with ECOG grade 0 (*n* = 20) showed superior survival outcomes, with 2‑ and 5‑year overall survival rates of 80.0 and 66.3%, respectively, and median survival not estimable due to insufficient events at final analysis. In contrast, ECOG grade 1 patients (*n* = 11) demonstrated notably shorter survival, with a median overall survival of 18.3 months (95% CI: 9.2–30.4), 2‑year survival of 28.3%, and no survivors at 5 years. The mortality rate in the ECOG grade 0 group was 30% (6 deaths), substantially lower than the 72.7% (8 deaths) observed in the ECOG grade 1 group (Fig. 4d).

### Impact of brachytherapy

**Fig. 5:**
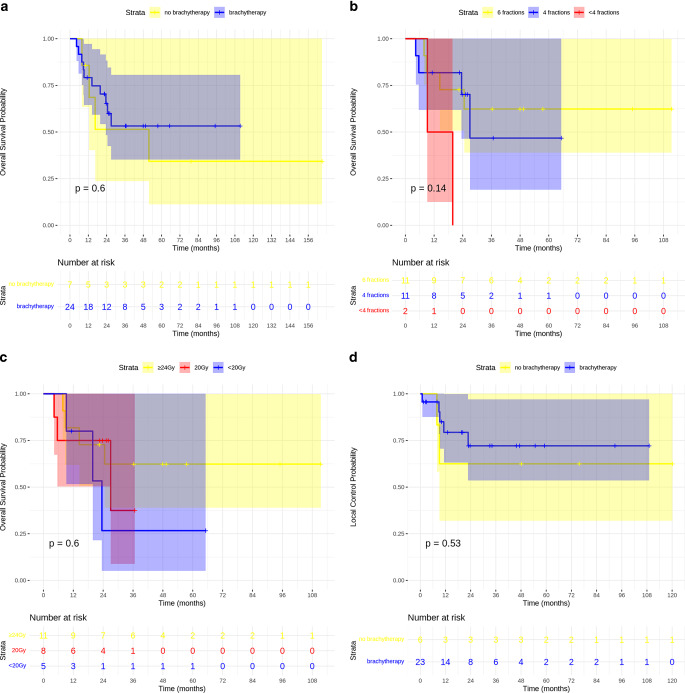
Impact of brachytherapy on overall survival (OS) and local control (LC): OS by brachytherapy application (**a**), OS by brachytherapy fractions (**b**), OS by brachytherapy total dose (**c**), LC by brachytherapy application (**d**)

Analysis of survival outcomes comparing brachytherapy (*n* = 24) and non-brachytherapy (*n* = 7) groups revealed differing mortality rates of 41.7% (10 deaths) and 57.1% (4 deaths), respectively (Fig. 5a). Overall survival probabilities at 2 years were 65% with brachytherapy versus 51% without, declining to 53 and 34%, respectively, at 5 years. Median overall survival was notably longer in the brachytherapy group (108.0 months) compared to the non-brachytherapy group (51.7 months; 95% CI: 16.2–126.8), although this difference did not reach statistical significance (log-rank test: *p* = 0.6).

Analysis of survival outcomes revealed a trend toward longer survival with increasing fraction number, albeit not reaching statistical significance (*p* = 0.14). The six-fraction group (*n* = 11) demonstrated the longest mean survival of 74.8 months (95% CI: 46.3–103.3), with median survival not estimable due to insufficient events at final analysis. This was followed by the four-fraction group (*n* = 11) with mean and median survival times of 40.4 (95% CI: 22.0–58.9) and 27.0 months, respectively. The shortest survival was observed in patients receiving fewer than four fractions (*n* = 2), with mean and median times of 14.5 (95% CI: 4.1–24.9) and 9.2 months, respectively (Fig. 5b).

Survival analysis stratified by the total brachytherapy dose demonstrated a trend toward improved outcomes with higher doses, albeit without statistical significance (*p* = 0.6). Patients receiving 24 Gy (*n* = 11) showed the most favorable outcomes, with a mean survival of 74.8 months (95% CI: 46.3–103.3) and median survival not reached. The 20-Gy group (*n* = 8) demonstrated a mean survival of 25.1 months (95% CI: 15.6–34.6) with median survival of 27.0 months, while patients receiving < 20 Gy (*n* = 5) showed a mean survival of 30.8 months (95% CI: 9.9–51.7) with median survival of 23.5 months (Fig. 5c).

Local recurrence rates were 21.7% (5/24) in the brachytherapy group versus 33.3% (2/6) in the non-brachytherapy group, with 2‑ and 5‑year local control (LC) rates of 72.1 versus 62.5%, respectively. Median time to local recurrence was not reached in either group, and no statistically significant difference in local control was observed between the groups (*p* = 0.53; Fig. 5d).

### Treatment-related toxicity

Of the 31 patients, 27 reported toxicities, with 83.95% of all recorded toxicity grades being grade 1 or 2. The most common symptoms included gastrointestinal issues (44.4%) and urogenital problems such as non-infectious cystitis (51.6%). Severe toxicities (grade 3–4) were infrequent, affecting only a small subset of patients, and primarily included anemia (19.4%) and fistula deterioration (16.1%). Among the 5 patients who developed post-therapeutic fistulas, two had received 1.6 Gy per fraction during EBRT, and 3 had received 1.8 Gy per fraction. Notably, all fistulas occurred after the completion of EBRT. Late toxicities were reported in 45.2% of patients, with most being mild to moderate (grade 1–2). The most common grade 1–2 late effects included proctitis or colitis (16.1%), pelvic insufficiency fractures (9.7%), and vaginal dryness (9.7%). Severe late toxicities (grade 3) were rare, affecting isolated cases. A detailed breakdown of toxicities by grade and symptom type is provided in Table 3.

**Table 3:** Post-treatment adverse events, categorized according to CTCAE version 5.0 [1]

Grade	0, % (*n*)	1, % (*n*)	2, % (*n*)	3, % (*n*)	4, % (*n*)
*Acute toxicity*					
Non-infectious cystitis	48.4 (15)	41.9 (13)	9.7 (3)	–	–
Diarrhea	61.3 (19)	22.6 (7)	12.9 (4)	3.2 (1)	–
Nausea	71 (22)	22.6 (7)	6.5 (2)	–	–
Erythema	71 (22)	22.6 (7)	3.2 (1)	3.2 (1)	–
Fatigue	93.5 (29)	3.2 (1)	3.2 (1)	–	–
Leukopenia	87 (27)	3.2 (1)	9.7 (3)	–	–
Anemia	58.1 (18)	16.1 (5)	6.5 (2)	19.4 (6)	–
Thrombocytopenia	93.5 (29)	3.2 (1)	3.2 (1)	–	–
Creatinine	90.3 (28)	9.7 (3)	–	–	–
Pain	87 (27)	9.7 (3)	3.2 (1)	–	–
Hearing impairment	96.8 (30)	–	3.2 (1)	–	–
Fistula deterioration	83.9 (26)	–	–	–	16.1 (5)
*Late toxicity*					
Proctitis/colitis	80.6 (25)	16.1 (5)	–	3.2 (1)	–
Urinary incontinence	87 (27)	13 (4)	–	–	–
Fecal incontinence	96.8 (30)	3.2 (1)	–	–	–
Pelvic insufficiency fracture	87 (27)	6.5 (2)	3.2 (1)	3.2 (1)	–
Vaginal dryness	90.3 (28)	6.5 (2)	3.2 (1)	–	–
Radiation erythema	93.5 (29)	3.2 (1)	3.2 (1)	–	–

In general, the observed toxicity profile aligns with expectations for definitive chemoradiotherapy in locally advanced cervical cancer. Most toxicities were manageable, and severe complications such as fistula formation (appearing at a median time of 5.7 weeks after the initiation of therapy, range 1–7 weeks) were addressed with appropriate interventions based on their severity, including surgical management for complex cases.

### Patient-reported outcomes (PROs)

Patient-reported outcome assessment was performed using a paper-based version of the EORTC QLQ-C30 and QLQ-BR23 questionnaires. In the domain of endocrine function, several patients reported a reduction in libido, with a few describing the symptoms as severe (grade 3). Most respondents indicated that they were not sexually active and reported low levels of interest and satisfaction in this area. Vaginal dryness was noted but did not cause significant discomfort. Physical well-being was largely characterized by mild to moderate impairments. Fatigue, pain, and limitations in daily activities such as work and hobbies were commonly reported, with 1 patient describing these issues as severe. Shortness of breath and physical limitations during exertion were less frequent but present in some cases. Emotional well-being was also affected, with occasional reports of irritability, worry, or sadness. Social and financial challenges were mentioned by a small number of patients. Regarding overall health and quality of life, most patients rated their status as “excellent,” while others reported notable impairments.

## Discussion

Our study highlights the pivotal role of advanced radiotherapy techniques for achieving durable LC in FIGO stage IVA cervical cancer. In our cohort, the 10-year LC probability was 70.4%, aligning with prior studies such as those by Schiff et al. and Park et al., which underscore the efficacy of BT in improving outcomes for advanced-stage disease [10, 11]. The significant correlation between LC and OS (*p* = 0.0039) further demonstrates the necessity of effective local therapies for improving long-term outcomes. However, the modest 5‑year OS rate of 46.2% reflects the high risk of systemic progression in this patient population.

The EMBRACE-I study, a pivotal investigation in MRI-guided adaptive brachytherapy, analyzed 1341 patients from 24 centers in Europe, Asia, and North America. Among these, only 34 patients had FIGO stage IVA disease, representing 2.5% of the cohort [12]. While the reported 5‑year LC rate of 91% and pelvic control rate of 81% for this subgroup are remarkable, the relatively small sample size, especially when compared to the overall cohort, necessitates cautious interpretation. In contrast, our study demonstrated a slightly lower 5‑year LC rate of 70.4% for FIGO stage IVA. This discrepancy could be attributed to several factors, including differences in CTVs. EMBRACE‑I reported a median high-risk CTV of 57 ccm (range 39–89), which reflects smaller target volumes. In contrast, our cohort had substantially larger target volumes, with a mean CTV for BT of 149 ccm (range 52–260 ccm). Another notable difference concerns the inclusion criteria with respect to paraaortic lymph node involvement. EMBRACE‑I restricted paraaortic nodal disease to metastases below the L1–L2 interspace, whereas our cohort included patients with more extensive paraaortic disease extending above the L2 level. Such extended nodal involvement is associated with poorer prognoses and may explain the observed differences in outcomes. Another significant factor is treatment duration. EMBRACE‑I reported a median overall treatment time (OTT) of 46 days (range 42–50 days), adhering closely to established guidelines for minimizing delays. In comparison, our median OTT was 69 days (range 36–105 days), reflecting delays that are well documented to reduce the efficacy of radiotherapy. While Shaverdian et al. suggest that delays may have less impact in concurrent chemoradiotherapy than in radiation monotherapy [13], minimizing interruptions remains essential for optimizing outcomes. The EMBRACE-II study builds on these findings, emphasizing the need for advanced techniques including prophylactic paraaortic irradiation and systematic use of concurrent chemotherapy [14].

Beyond these established approaches, recent advances in radiation oncology have introduced online adaptive radiotherapy (online ART) as a promising tool to further enhance precision and minimize toxicity [15]. Online ART enables real-time modification of treatment plans based on daily anatomical changes, thereby potentially improving target coverage while reducing radiation exposure to adjacent OARs [16]. Peng et al. conducted a dosimetric evaluation of online ART in uterine cervical cancer and demonstrated improved target volume conformity and reduced dose exposure to the rectum, bladder, and small intestine [17]. In addition to dosimetric benefits, Jiang et al. provided the first clinical data on online ART in cervical cancer, demonstrating that this technique can significantly enhance PTV coverage while reducing doses to OARs [18]. However, its application in advanced FIGO stage IVA cervical cancer with bladder and rectal infiltration remains largely unexplored. Unlike in earlier-stage disease, where online ART allows for margin reduction to spare OARs, the risk of marginal underdosage must be carefully considered in FIGO IVA cases, where adequate dose escalation is critical for local control. Despite these advantages, online ART still faces practical limitations, including longer treatment times, high resource demands, and limited clinical validation in advanced-stage disease. Furthermore, while early studies indicate reduced OAR toxicity, robust clinical data on long-term oncological outcomes and toxicity profiles are still lacking. Prospective trials are needed to determine whether online ART can serve as a viable tool for balancing dose escalation and toxicity reduction in high-risk patients.

Our findings reaffirm the pivotal role of BT in improving outcomes, particularly when integrated with concurrent chemoradiotherapy. Park et al. demonstrated superior LC rates in patients with bladder or rectal invasion treated with BT compared to those receiving only an EBRT boost [11]. Mazeron et al. and Pötter et al. further emphasized the importance of dose escalation in BT for achieving optimal LC [12, 19]. Consistent with these findings, our cohort showed that patients receiving six fractions or ≥ 24 Gy of BT exhibited the most favorable outcomes, with 5‑year OS probabilities of 62.3%. The estimated median OS was 108 months for patients treated with BT and 51.7 months for those without, highlighting the survival advantage associated with BT. Although these differences were not statistically significant, they align with prior studies associating higher doses to improved tumor control. These results reinforce the integral role of high-dose-rate BT in the definitive treatment of advanced cervical cancer. Larger prospective studies are necessary to validate these trends and to determine precise dose thresholds to optimize both LC and survival.

Prognostic factors play a substantial role in determining survival outcomes. Our study reaffirmed the significant prognostic value of ECOG performance status (*p* = 0.014), with superior outcomes observed in patients with ECOG 0 compared to ECOG 1. Although patient age did not reach statistical significance (*p* = 0.3), a clear trend toward improved outcomes in younger patients (< 50 years) was observed, consistent with prior studies [20, 21]. Advanced tumor size (≥ 6 cm), pelvic lymph node involvement, and paraaortic lymph node metastasis have been shown to negatively impact survival [11]. Comprehensive nodal assessment during staging is critical for identifying high-risk patients who may benefit from intensified systemic therapies or closer post-treatment surveillance.

While achieving robust LC remains a primary therapeutic goal, the predominance of distant failures highlights the need for systemic therapies to improve OS. Immune checkpoint inhibitors such as pembrolizumab have shown significant promise in this context. The KEYNOTE-A18 trial demonstrated a 30% reduction in the risk of disease progression or death with the addition of pembrolizumab to chemoradiotherapy, with 2‑year PFS rates of 68–57% for chemoradiotherapy alone, particularly in PD-L1-positive and HPV-driven tumors. The trial further highlighted that pembrolizumab may offer particular benefit in patients with high-risk disease, including those with nodal involvement [22]. Similarly, induction chemotherapy (NACT) has garnered interest for its potential to improve outcomes by reducing tumor burden and addressing micrometastatic disease prior to definitive chemoradiotherapy. The INTERLACE trial demonstrated a significant improvement in PFS (5-year PFS: 72 vs. 64%) and OS (5-year OS: 80 vs. 72%) with short-course induction chemotherapy followed by chemoradiotherapy compared to chemoradiotherapy alone [23]. However, the study cohort primarily consisted of stage IIB patients, with limited representation of stage IVA disease and exclusion of paraaortic nodal involvement, thus raising questions about the generalizability of these findings to more advanced cases. Additionally, the OUTBACK trial evaluated the role of adjuvant chemotherapy following standard chemoradiotherapy [24]. Despite the hypothesis that adjuvant chemotherapy might reduce distant metastases, the trial found no improvement in 5‑year OS (72% with adjuvant chemotherapy vs. 71% without), while highlighting an increase in treatment-related toxicity. These results suggest that additional chemotherapy after chemoradiotherapy may not provide sufficient benefit to justify the associated risks. Collectively, these findings underscore the need for further prospective studies to optimize systemic therapy approaches in patients with advanced-stage disease, particularly those with bladder or rectal infiltration, and to refine patient selection for immunotherapy and induction chemotherapy.

In parallel, advancements in liquid biopsy technology could transform the therapeutic landscape. Circulating tumor DNA (ctDNA) and circulating tumor cells (CTCs) provide opportunities for early detection, treatment monitoring, and recurrence identification [25, 26]. Specific biomarkers, such as cell-free HPV DNA (ctHPV DNA), show promise as prognostic indicators, correlating with advanced disease stages and poorer outcomes [27]. However, their widespread implementation faces challenges, including standardization of preanalytical processes and cross-platform validation [28–30]. Despite these limitations, liquid biopsy represents a significant step toward precision medicine, offering a non-invasive approach to personalized cancer care.

Management of treatment-related toxicities remains a critical component of optimizing cervical cancer treatment. Acute toxicities were observed in 87.1% of patients in our cohort, with the most common gastrointestinal symptoms being diarrhea (38.7%) and nausea (29.1%). Among urogenital toxicities, non-infectious cystitis was reported in 51.6% of patients and was the most common toxicity in this category. These findings are consistent with previous studies by Maduro et al. and Alfrink et al., which reported gastrointestinal, hematological, and urogenital toxicities as common side effects of chemoradiotherapy [31, 32]. Severe acute toxicities (grade 3–4) were rare, occurring in only 13 cases, consistent with findings from Schiff et al. [10]. Our findings concerning late toxicities are consistent with those of Pötter et al., who reported that grade 1 and 2 late toxicities constituted the majority of adverse effects, while severe complications were infrequent [12]. In our cohort, late toxicities were observed in 45.2% of patients, with the most common being radiation proctitis/colitis (19.3%) and urinary incontinence (13%). Pelvic insufficiency fractures occurred in 12.9%, while fecal incontinence was observed in 3.2% of cases. These complications can significantly impact long-term quality of life, affecting bowel, bladder, and musculoskeletal function. Given their potential impact on daily living, these late effects should be actively considered in survivorship care planning, including early intervention strategies and long-term supportive care programs.

Fistula formation remains a particularly severe late complication, occurring in 16.1% of our cohort. This aligns with reported rates in the literature, which range from 4.5 to 44%, depending on patient characteristics and treatment protocols [33–35]. Risk factors include tumor progression, prior pelvic radiation, invasive procedures, and high radiation doses to adjacent organs and the use of bevacizumab in combination with chemoradiotherapy [36–38]. Hata et al. have suggested that fistula development in cases of pre-treatment organ infiltration may also indicate effective therapy, marked by tumor shrinkage and withdrawal from surrounding tissues [39]. Additionally, advanced tumor stage, older age, poor performance status, and high radiation doses to adjacent organs, particularly the rectum, have been highlighted as contributors [39]. However, intensified treatment regimens can heighten this risk, as noted by Biewenga et al. [40]. In response to these risks, modifications to radiation protocols have been proposed to minimize exposure to critical organs and reduce the likelihood of fistula formation. Hata et al. demonstrated that reducing fraction doses—administering 1.8 Gy per fraction in EBRT and 5 Gy per fraction in BT compared to the standard doses of 2 and 6 Gy, respectively—led to a significant reduction in vesicovaginal fistula rates, with only one case reported among 26 patients with bladder infiltration [39]. While further dose reductions, such as the 1.6 Gy per fraction used in selected patients in our cohort, may represent an additional step toward mitigating the fistula risk, our data suggest that the timing of and tumor response to EBRT, rather than BT or fraction size, may play a more critical role in fistula development. Prospective studies are needed to further explore these strategies and validate their effectiveness.

Beyond physical complications, the long-term impact on quality of life (QoL) is significant. Patients often experience a combination of physical and psychosocial challenges, including fatigue, diarrhea, urinary symptoms, anxiety, and depression [41]. These findings underscore the importance of integrating psychosocial support and survivorship programs into comprehensive cervical cancer care.

Finally, global disparities in access to advanced therapies, such as MRI-guided BT and modern imaging techniques, remain a major challenge, particularly in low- and middle-income countries [42]. International collaboration, investment in healthcare infrastructure, and the development of cost-effective technologies are essential to bridging these gaps and ensuring equitable access to high-quality cervical cancer care.

Several limitations of this study must be acknowledged. The small sample size, particularly in subgroup analyses (e.g., patients receiving fewer than four fractions of BT), limits the statistical power and generalizability of the findings. The retrospective design introduces potential biases, including variability in patient selection, incomplete follow-up, and inconsistencies in treatment delivery. Furthermore, the absence of standardized imaging protocols for post-treatment surveillance may have contributed to underreporting or misclassification of recurrences and metastases.

While this study underscores the importance of brachytherapy and concurrent chemoradiotherapy in FIGO stage IVA cervical cancer, prospective trials with larger cohorts are needed to confirm these findings. Future research should focus on refining therapeutic strategies for high-risk subgroups, such as those with advanced organ infiltration or lymph node involvement. Emerging techniques, such as liquid biopsy for treatment monitoring and recurrence detection and the integration of immunotherapy to improve systemic control, warrant further investigation. Additionally, advancements in adaptive radiotherapy and dose optimization could enhance local control while minimizing treatment-related toxicities.

## Conclusion

This study highlights the critical role of advanced radiotherapy techniques, particularly MRI-guided brachytherapy, in the management of FIGO stage IVA cervical cancer. These methods provide effective local control and reduce the need for invasive procedures such as pelvic exenteration. Despite the high risk of systemic progression, these approaches remain central to improving survival outcomes. Future studies should validate these findings in larger, multi-institutional cohorts and explore systemic therapies to address distant failures. Expanding global access to these advanced treatments is essential for improving outcomes in this high-risk population.
